# Experimental and Computational Studies Reveal Novel Interaction of Lymphocytes Antigen 6K to TGF-β Receptor Complex

**DOI:** 10.3390/ijms241612779

**Published:** 2023-08-14

**Authors:** Justyna Andrys-Olek, Benson Chellakkan Selvanesan, Sheelu Varghese, Ricardo Hernandez Arriaza, Purushottam Babu Tiwari, Maksymilian Chruszcz, Tomasz Borowski, Geeta Upadhyay

**Affiliations:** 1 Jerzy Haber Institute of Catalysis and Surface Chemistry, Polish Academy of Sciences, 30-239 Cracow, Poland; 2 Department of Pathology, Uniformed Services University of the Health Sciences, Bethesda, MD 20824, USA; 3 Henry M. Jackson Foundation, Uniformed Services University of the Health Sciences, Bethesda, MD 20814, USA; 4 Department of Biochemistry and Molecular Biology, Michigan State University, East Lansing, MI 48825, USA; 5 Department of Chemistry and Biochemistry, University of South Carolina, Columbia, SC 29208, USA; 6 Department of Oncology, Georgetown University Medical Center, Washington, DC 20007, USA; 7 John P. Murtha Cancer Center, Bethesda, MD 20814, USA

**Keywords:** LY6K, TGF-β signaling, receptor complex, TGF-β1

## Abstract

TGF-β signaling promotes migration, invasion, and distant colonization of cancer cells in advanced metastatic cancers. TGF-β signaling suppresses the anti-tumor immune response in a tumor microenvironment, allowing sustained tumor growth. TGF-β plays an important role in normal physiology; thus it is no surprise that the clinical development of effective and safe TGF-β inhibitors has been hampered due to their high toxicity. We discovered that increased expression of LY6K in cancer cells led to increased TGF-β signaling and that inhibition of LY6K could lead to reduced TGF-β signaling and reduced in vivo tumor growth. LY6K is a highly cancer-specific protein, and it is not expressed in normal organs except in the testes. Thus, LY6K is a valid target for developing therapeutic strategies to inhibit TGF-β signaling in cancer cells. We employed in vitro pull-down assays and molecular dynamics simulations to understand the structural determinants of the TGF-β receptor complex with LY6K. This combined approach allowed us to identify the critical residues and dynamics of the LY6K interaction with the TGF-β receptor complex. These data are critical in designing novel drugs for the inhibition of TGF-β in LY6K expressing cancer, induction of anti-tumor immune response, and inhibition of tumor growth and metastatic spread.

## 1. Introduction

TGF-β signaling is a major immune suppressive pathway in cancer progression, which can inhibit the expansion of cytotoxic T-cells and promote the exhausted phenotype of cytotoxic immune cells, leading to sustained cancer growth [1]. In addition to its effect on immune cells, increased TGF-β signaling in cancer cells can promote invasion and metastasis [2]. TGF-β signaling is propagated by the TGF-β receptor complex composed of TGF type-1 receptor (TbR1) and TGF type-2 receptor (TbR2) [3]. The homodimers of TbR1 and TbR2 form a tetramer in the presence of TGF-β ligands [3]. In this complex, TbR2, a constitutively active kinase, phosphorylates TbR1, which in turn phosphorylates intracellular Smad2/3 protein [3]. A phosphorylated Smad2/3 forms a heterodimer with Smad4 protein that can translocate to the nucleus and participate in a transcriptional program to exert downstream effects of TGF-beta signaling [3]. The regulation of the TGF-β receptor complex is a dynamic process tightly controlled by the TGF-β ligand. The deregulation of this signaling complex occurs in metastatic cancers and in many kinds of solid cancers, where TGF-β drives tumor cell migration and invasion [4]. In addition to its direct effect on cancer cells, increased TGF-β diminishes the infiltration of the cytotoxic T-lymphocytes (CTLs) and natural killer (NK) cells into the tumor microenvironment and promotes the expansion of suppressive immune cells, including T-regulatory (T-regs) cells and myeloid-derived suppressive cells (MDSC) [4,5]. These cumulative effects contribute to sustained tumor growth and metastasis.

The inhibition of TGF-β signaling is a major target for developing anti-cancer drugs due to the prominent role of TGF-β signaling in metastatic tumor growth [6]. Since TGF-β signaling is essential for normal cellular function, a general inhibition of TGF-β signaling induces significant systemic side effects, leading to the halt of such treatments [7]. Thus, it is desirable to seek tumor-specific modifiers of TGF-β signaling that can be targeted for novel therapeutics aimed at inhibiting TGF-β signaling specifically in tumors and their microenvironments. We reported that lymphocyte antigen-6 (LY6K) is required for increased TGF-β signaling, thereby contributing to the in vivo growth of triple-negative breast cancer cells [8]. LY6K is a small glycoprotein that belongs to the LU domain family of proteins, which includes stem cell antigen-1 (Sca-1). We found that Sca-1 binds to TGF-β receptor 1 (TbR1) to disrupt TGF-β signaling [9]. Sca-1 is the first identified member of the mouse LY6 family of proteins residing on chromosome 15, which is not found in humans [10]. The human LY6 family of proteins including LY6K reside in the syntenic area of chromosome 8, and they are upregulated in many types of human cancers, including, breast, brain, lung, ovarian, bladder, kidney, and head and neck cancer [11,12]. We investigated whether the human LY6K facilitates TGF-β signaling through its interaction with TbR1. In this report, we show that LY6K binds to TbR1 and provides structural insights into this interaction. The structural insight into the LY6K-TbR1 interaction may shed light on strategies for disrupting TGF-β signaling in cancer cells expressing LY6K. 

## 2. Results

### 2.1. Direct Interaction of LY6K and TbR1

**Figure 1:**
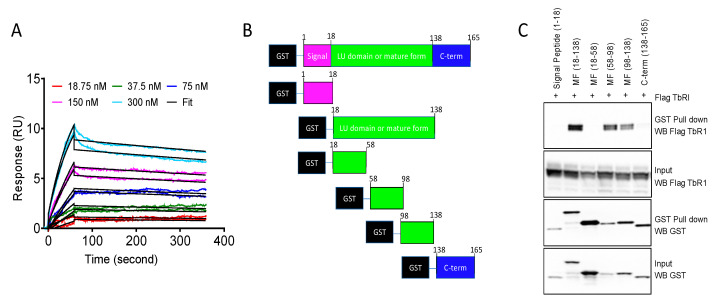
LY6K interacts with TbR1. (**A**) SPR sensorgrams for TbR1 (at various concentrations) binding to immobilized LY6K-Wt onto a CM5 chip surface. The colored lines are the experimental data. A K_D_ value of 9.2 nM was obtained from the fitting (black lines) of the experimental data (colored lines) to the 1:1 kinetics binding model, k_a_ (1/Ms) = 5.099 × 10^4^; k_d_ (1/s) = 0.0004682, and Ch^2^ = 0.0321. The SPR experiment was performed twice, with two technical replicates in each run. (**B**) GST-tagged full-length or deletion constructs of LY6K were cloned for mammalian expression. (**C**) The indicated GST-tagged LY6K proteins were co-expressed with flag-tagged TbR1 in 293T cells. LY6K fragments 58–98 or 98–138 were sufficient and required to bind to TbR1. The GST pull-down experiment was performed three times.

To test whether LY6K and TbR1 proteins interact directly with each other, surface plasmon resonance was applied to the purified proteins. We observed a direct binding of purified recombinant protein TbR1 to LY6K in a concentration-dependent manner with a K_D_ value of 9.2 nM, k_a_ (1/Ms) = 5.099 × 10^4^, k_d_ (1/s) = 0.0004682, and Ch^2^ = 0.0321 (Figure 1A).

We used GST-tagged deletion constructs of LY6K to determine the LY6K residues interacting with TbR1 (Figure 1B). We observed that LY6K residues 58–138, located in the LU domain, were sufficient for binding to TbR1. LY6K residues 18–58, located in the LU domain did not interact with TbR1. LY6K signal peptide (1–18) and pro-peptide (138–168) did not interact with TbR1 (Figure 1C).

### 2.2. Docking LY6K Models to TGF-β Complex in HADDOCK

**Figure 2:**
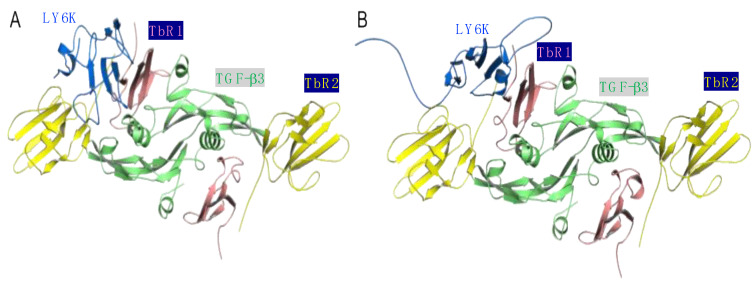
Results of docking LY6K model (blue) to the TGF-β dimer complex (**A**) I-TASSER LY6K model as a ligand. (**B**) AlphaFold2 LY6K model as a ligand. Colored as follows: TbR1 in pink, TbR2 in yellow, and TGF-β3 ligand in green.

The structure of the I-TASSER model of LY6K in the complex with the TGF-β dimer complex chosen from the docking was the second-best complex ranked by HADDOCK with a score of 132.1 ± 13.1 (the best one had a score of 103 ± 14.1) and came from the cluster of size 4 (Figure 2A). Notably, the first and second-best docking pose had similar binding energies calculated using the MMPBSA method (53.96 ± 15.53 kcal/mol for the best vs. 58.85 ± 16.37 kcal/mol for the second best) as well as comparable docking score and cluster sizes (6 and 4 for the first and second-best pose, respectively). We chose the second-best pose, which was in close agreement with the pull-down data (Figure 1) showing the interaction of LY6K residues 98–138 with TbR1. On the other hand, the structure of the AlphaFold2 model of LY6K in complex with the TGF-β dimer chosen from the docking was the best complex ranked by HADDOCK with a score of 280.8 ± 5.0, which came from the cluster of size 6 (Figure 2B). Please note that the HADDOCK score should not be used as an estimate of protein–protein interaction energy.

### 2.3. Modeling LY6K-TbR1 Complex

**Figure 3:**
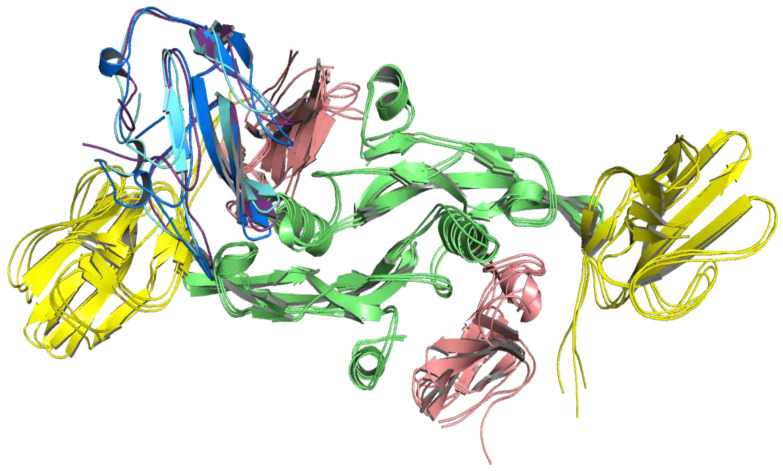
MD simulation for LY6K (monomer) I-TASSER model docked to TGF-β receptor complex (dimer). Results of clustering frames from a 100 ns long molecular dynamics simulation into three clusters: LY6K geometry from the largest cluster shown in dark blue, from the second-largest cluster shown in light blue, and from the third cluster shown in purple. TbR1 in pink, TbR2 in yellow, and TGF-β3 ligand in green.

For the molecular dynamics simulation, the geometries of the LY6K-TGF-β receptor complexes (dimers) for both LY6K models (I-TASSER and AlphaFold2) served as input structures. Then, they underwent 100 ns of molecular dynamics simulations. After visual inspection of the trajectories, the frames were clustered into three clusters, based on the mutual orientation between LY6K and the TGF-β complex dimer, to analyze the stability of the interaction. In the case of the complex with the I-TASSER LY6K model, the largest cluster covered over 55% of the trajectory, the second-largest covered over 24%, and the third-largest covered approximately 21% (Figure 3).

**Figure 4:**
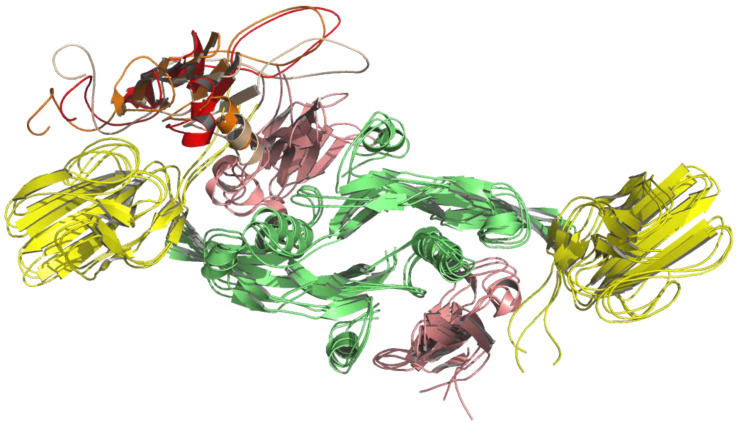
MD simulation of LY6K (monomer) AlphaFold2 model docked to the TGF-β receptor complex (dimer). Results of clustering frames from a 100 ns long molecular dynamic simulation into three clusters: LY6K geometry from the largest cluster shown in red, from the second-largest cluster shown in orange, and from the third cluster shown in light orange. TbR1 in pink, TbR2 in yellow, and TGF-β3 ligand in green.

In the case of the complex with the AlphaFold2 LY6K model, the largest cluster covered approximately 59% of the trajectory, the second-largest covered around 34%, and the third cluster covered approximately 7% (Figure 4). In both simulations, the differences between centroids (representative geometries) of the three clusters were rather subtle (Figure 3 and Figure 4) and were mostly the result of the mobility of the loop regions in the ligand (LY6K). MMPBSA energy calculations performed for the dominant (most stable) cluster of MD frames revealed that the TGF-β dimer in the complex with the AF2 or I-TASSER model had comparable Poisson–Boltzmann energies (43.80 ± 19.65 kcal/mol and 58.85 ± 16.37 kcal/mol, respectively).

**Figure 5:**
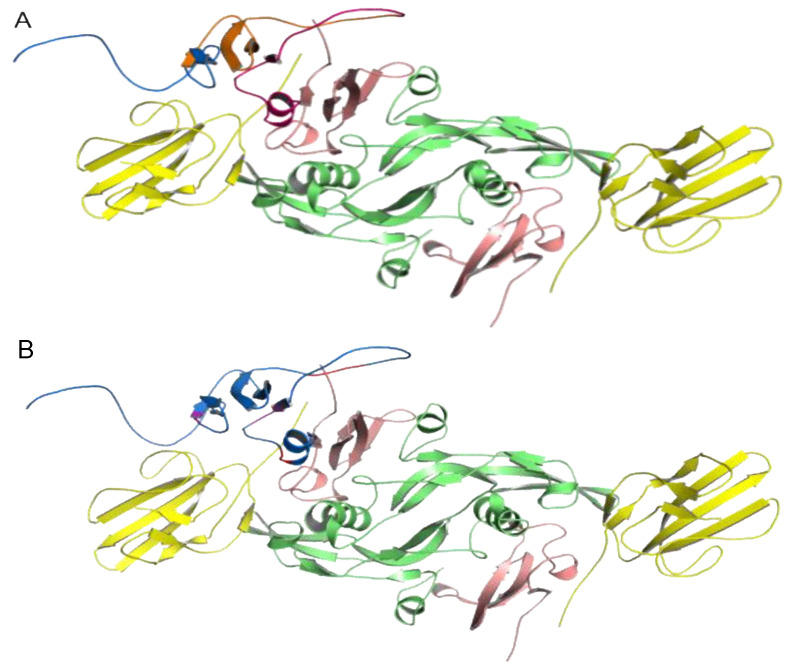
Native contacts analysis of the I-TASSER model of the LY6K—TGF-β complex. (**A**) LY6K protein is colored as follows: 18–57 aa in blue; 58–98 aa in orange; 99–138 in magenta. (**B**) LY6K protein colored according to native contact analysis: red indicates residues interacting with TbR1; light blue indicates residues interacting with TbR2. All of them are in the 58–98 aa range. TbR1 in pink, TbR2 in yellow, and TGF-β3 ligand in green.

**Figure 6:**
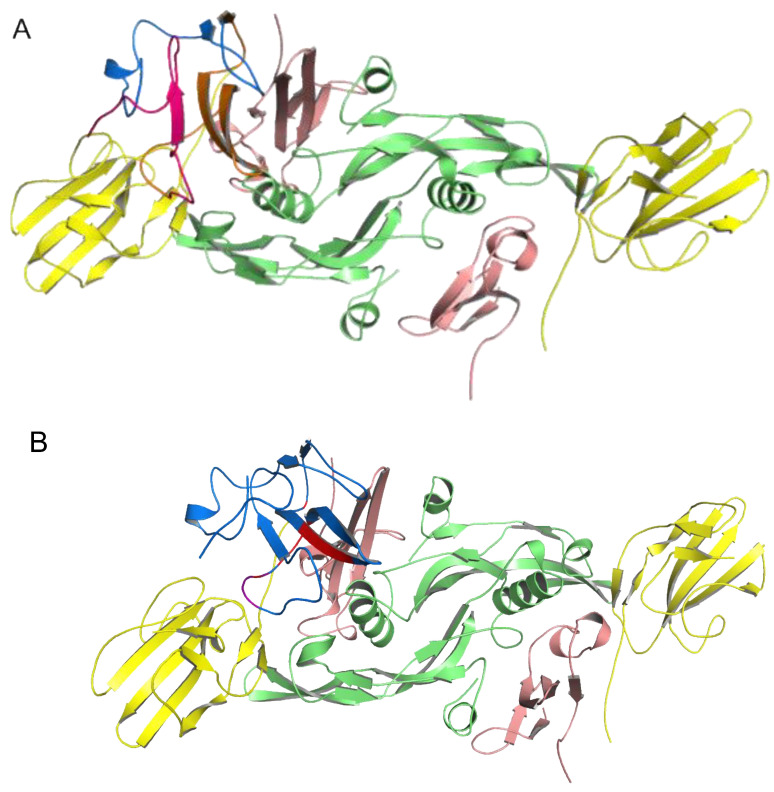
Native contacts analysis of the AlphaFold2 model of the LY6K—TGF-β complex. (**A**) LY6K protein is colored as follows: 18–57 aa in blue; 58–98 aa in orange; 99–138 in magenta. (**B**) LY6K protein colored according to native contact analysis: red indicates residues interacting with TbR1; purple indicates residues interacting with TbR2. TbR1 in pink, TbR2 in yellow, and TGF-β3 ligand in green.

To find out which LY6K residues were interacting with the TGF-β complex (more importantly, with TbR1), a native contact analysis was performed. The interfaces of the LY6K-TbR1 interaction differed between the models, but in both cases, they involved residues from the 58–98 fragment of LY6K. The I-TASSER model interacted with TbR1 via residues 63, 70–74, 86–89, and 92, and with TbR2 via 91 and 93–95 (Figure 5). The AlphaFold2 model interacts with TbR1 via residues 91–96 and 127–130, and with TbR2 via residues 55–57, 92–94, and 113–121 (Figure 6).

## 3. Discussion

In this study, we showed that one of the underlying mechanisms of LY6K in increasing TGF-β signaling could be via its direct interaction with the TGF-β receptor complex. The results of SPR and pull-down studies suggest that LY6K directly binds at least to the TbR1 protein in the TGF-β receptor complex. Interestingly, we observed a good agreement between GST pull-down studies and MD modeling, showing that amino acids 58–138 of LY6K may be directly involved in the interaction with TbR1. Both I-TASSER and AlphaFold2 modelling confirmed that the 58–98 fragment of LY6K interacts with TbR1. The AlphaFold2 model showed that amino acids 127–130 of LY6K interact with TbR1. Both I-TASSER and AlphaFold2 models showed that LY6K interacts with TbR2 in this complex via amino acids 91, 93–95, 55–57, 92–94, and 113–121 (Table 1). These analyses indicate that common residues on LY6K may interact with both TbR1 and TbR2.

Future studies should focus on strategies to disrupt the LY6K-TGF-β receptor complex to attenuate this signaling in cancer cells. While this manuscript was in progress we were happy to see that LY6K and TbR1 interacted in cancer cells [13]. These results confirm our previous observation that members of the LY6 protein family may interact with TbR1 to modulate TGF-β signaling [9]. The data presented in this report elaborate on amino acid residues in LY6K, which may interact with TbR1 to modulate the TGF-β receptor complex in cancer cells and affect the tumor microenvironment. We have discovered that a small molecule binder of LY6K can inhibit LY6K-signaling, reduce tumor growth, and induce an anti-cancer immune response in vivo [14,15]. Future studies should focus on novel inhibitors of LY6K that can inhibit TGF-β signaling in an LY6K-specific manner to develop novel drugs with improved specificity, efficacy, and reduced toxicity.

## 4. Methods and Material

### 4.1. Surface Plasmon Resonance

Recombinant human TbR1 protein was purchased from R&D Systems, Inc. Minneapolis, MN, USA. The cDNA encoding human LY6K protein was cloned into the pET24 vector and recombinant protein was purified using a nickel column. The Biacore T200 instrument was used to record SPR sensorgrams at 25 °C. LY6K protein was immobilized on a CM5 chip using the standard amine coupling method in a 10 mM sodium acetate buffer at pH 4.0 within flow cell (FC) 2, leading to a level of ~6200–7000 RU. FC1 was used as the reference FC, which had the same surface chemistry as FC2, but no proteins were immobilized on it. PBS-P (20 mM phosphate buffer pH 7.4, 2.7 mM KCl, 137 mM NaCl, and 0.05% surfactant P20) was used as the immobilization running buffer. Various concentrations of TbR1 were injected into the reference (FC1) and active (FC2) FCs at a flow rate of 50 μL/min. The association and dissociation times were 60 s and 300 s, respectively. Each concentration of TbR1 was injected in triplicate. PBS-P + 5% DMSO was used as the running buffer for the TbR1-LY6K interactions. One 20 s pulse of 1 M NaCl was injected for surface regeneration. The SPR sensorgrams were both the reference (signals corresponding to FC1) and blank (running buffer only) subtracted. The resulting sensorgrams corresponding to LY6K were evaluated using Biacore T200 Evaluation Software version 1.0 to determine K_D_ values for TbR1 bindings to the LY6K.

### 4.2. Plasmids

Human LY6K cDNA was cloned in pEBG GST vector in the indicated frames to be expressed in mammalian cells. The flag-tagged TbRI construct was a gift from Dr. Rik Derynck, UCSF, CA, USA.

### 4.3. Cell Culture and Transfection

HEK 293 cells (American Type Culture Collection, Manassas, VA, USA) were cultured in DMEM medium containing 10% FBS and 1× P/S antibiotics (Thermo Fisher Scientific, Waltham, MA USA). Cells were transfected with indicated expression constructs using lipofectamine 2000 (Thermo Fisher Scientific, Waltham, MA, USA).

### 4.4. GST Pull Down, Western Blotting Experiments

For GST pull-down, cell lysates were prepared in the 1× RIPA lysis buffer (Sigma-Aldrich, Inc., St. Louis, MO, USA) in the presence of 1× Halt™ Protease and Phosphatase Inhibitor (Thermo Fisher Scientific, Waltham, MA, USA) according to the product instructions. The GST-tagged LY6K protein was eluted using Glutathione Sepharose 4B GST beads (Cytivia, Marlborough, MA, USA) as indicated in the product instructions. The pull-down and input lysates were separated using a 4–12% gradient gel (Thermo Fisher Scientific, Waltham, MA, USA) and transferred using a trans-blot turbo transfer system (Bio-Rad Life Science, Hercules, CA, USA). Western blotting was performed using rabbit polyclonal Flag antibody (Cat #8146, Cell Signaling Technologies, Danvers, MA, USA), mouse monoclonal GST antibody (Cat #sc-138, Santa Cruz Biotechnology, Inc., Dallas, TX, USA), and HRP-tagged rabbit and mouse secondary antibodies (Cell Signaling Technologies, Danvers, MA, USA). The chemiluminescent signals were developed using SuperSignal™ West Pico PLUS Chemiluminescent Substrate and visualized using an iBright FL 1500 imaging system (Thermo Fisher Scientific, Waltham, MA, USA).

### 4.5. Molecular Dynamics Models

#### Preparation for Docking

**Figure 7:**
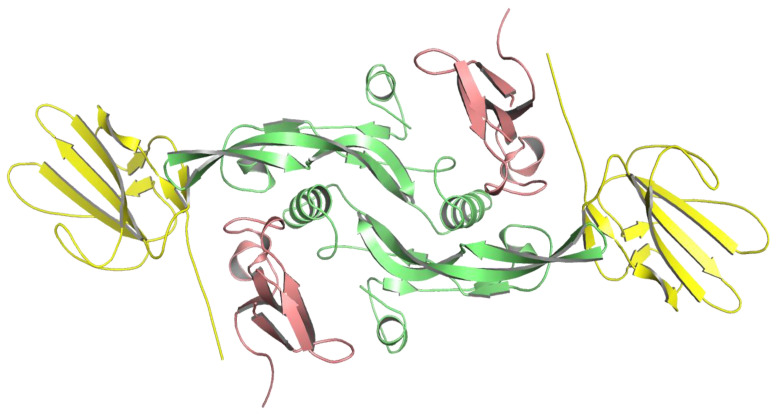
Crystal structure of TGF-β receptor complex (PDB: 2PJY) in dimeric form, colored as follows: TbR1 in pink; TbR2 in yellow, and TGF-β3 ligand in green.

To create a model of LY6K in complex with the TGF-β receptor complex, the HADDOCK2.4 server was used [16]. For docking purposes, the crystal structure of the TGF-β receptor complex in dimeric form (PDB: 2PJY, 3 Å resolution) was used (Figure 7) [17]. One monomer consists of two TGFβ-β receptors, namely TGF-β type 1 receptor (TbR1) and TGF-β type 2 receptor (TbR2) and its ligand TGF-β3 ligand. Although there is another crystal structure of TGF-β receptor complex available in PDB (PDB: 3KFD), captured with the same 3 Å resolution [18], the advantage of using 2PJY is that in the 2PJY crystal structure, the loop 64–68 in TbR1, a region believed to undergo a random coil—α-helix transition, is partially stabilized (the helix is formed), as opposed to the 3KFD structure, in which this fragment is destabilized (it remains in the coil conformation). Because the primary aim of this paper is to describe the interactions between the TbR1 and LY6K, a more ordered crystal structure was chosen. To assess the protonation state at pH 7.0, the crystal structure was processed using the propKa program, which is part of the ProteinPrepare program available on the PlayMolecule server [19].

**Figure 8:**
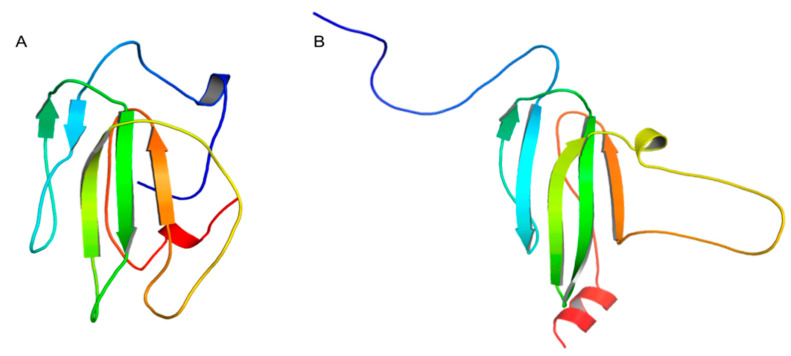
Computational 3D models of the mature form of LY6K structure: (**A**) LY6K I-TASSER model; (**B**) LY6K AlphaFold2 model. Protein chain in rainbow colors: N-termini in blue; C-termini in red.

As a ligand for docking, a theoretical LY6K protein model was used [14]. Briefly, two models of the mature form of the LY6K protein (amino acids: 18–138) were created. The first model was obtained using the I-TASSER server [20]. Then, after the analysis of 76 ns long molecular dynamics trajectory and clustering based on RMSD for the protein backbone, the centroid of the most populated cluster served as a model for further modeling steps (Figure 8A). A second alternative model of mature LY6K was created using the AlphaFold2 algorithm [21]. The three-dimensional output model ranked as the best prediction underwent a 100 ns long MD simulation. Then, once again an analysis of trajectory and frame clustering was performed to select the most stable protein geometry, with the centroid of the most populated cluster serving as the LY6K model (Figure 8B). Both LY6K models were then processed with the propKa program, setting pH 7.0, via the ProteinPrepare program available on the PlayMolecule server [19]. The two LY6K models differ in conformation, mainly in the N- and C-termini and the loop fragment, but at present, the biological relevance of these differences can be assessed only indirectly via comparison of computational and experimental data.

### 4.6. HADDOCK Docking

For docking purposes, the HADDOCK2.4 (High Ambiguity Driven protein–protein DOCKing) server was used. During the docking procedure, the TGF-β receptor complex (2PJY) in a dimeric form was set as a receptor, whereas one of the LY6K models served as a ligand. The HADDOCK scoring function is a combination of various energies (e.g., van der Waals, electrostatics, distance restraints energy, and desolvation energy) and buried surface area. The lower the score, the more accurate the result is believed to be. Apart from the HADDOCK scoring, the second criterion considered when selecting the best pose came from the experimental data, as the span of LY6K residues interacting with TbR1 in the complex was compared to the results of the GST pull-down experiment.

### 4.7. Molecular Dynamics Simulations

Models of TGF-β complexes with the LY6K model (either I-TASSER or AF2) were placed in a simulation box of a size such that its walls were at least 10 Å away from the protein atoms. Then, the box was filled with TIP3P water molecules and Na^+^/Cl^−^ ions, the latter in a number such that the whole system was charge neutral and the ionic strength was 0.15 M, which is considered to be a physiological salt concentration. In the case of the AF2 model of LY6K with the TGF-βreceptor complex, 46,781 water molecules, 114 chloride ions, and 126 sodium ions were added to the box. In the case of the I-TASSER model of LY6K with the TGF-β receptor complex, 29,256 water molecules, 87 chloride ions, and 99 sodium ions were added. Then, both models underwent the same procedure of MD simulation. First, three consecutive steps of energy minimization were performed: solvent relaxation with a protein complex restrained with a force constant of 500 kcal/mol·Å^2^, solvent relaxation with a protein complex restrained with a force constant of 10 kcal/mol·Å^2^, and solvent and protein relaxation without any restraints. Then, the system was subjected to heating from 0 to 300 K during 0.1 ns long NV dynamics with a Langevin thermostat. Before entering the 100 ns long production phase of the simulation, the system was equilibrated at a constant pressure of 1 bar at a temperature of 300 K. Such conditions were also set for the production phase of the simulation, which was run using the AMBER’s pmemd program [22]. The geometries were saved every 5000 steps, and the step size was 2 fs, as the SHAKE algorithm was applied to the bonds and angles involving hydrogen atoms.

### 4.8. Molecular Dynamics Simulations Analysis and MMPBSA Energy Calculation

The Cpptraj program from the AmberTools package was used to analyze the trajectories and gather protein–protein interaction data [22]. For clustering the geometries from the MD trajectory, based on structural similarity in a region where LY6K interacts with the TGF-β complex, the HierAgglo (hierarchical agglomerative) algorithm was applied. Then, for determining the LY6K amino acid residues interacting with the TGF-β complex (TbR1 in particular), native contact analysis was performed. After clustering, the MMPBSA energy was calculated for the most dominant cluster covering the parts of the MD trajectory in which the LY6K-TGF-β complex interaction was stable. The MMPBSA energy was calculated to assess the strength of the protein–protein binding.

## Figures and Tables

**Table 1:** A summary of amino acid (aa) residues on LY6K, which interact with TbR1 and TbR2 in two different computational models.

Identification of Amino Acid Residues on LY6K which Interact with TGF-β Receptor Complex Components			
Modelling	TbR1	TbR2	ΔE_PB
AlphaFold2 method and interacting LY6K amino acid residues (aa)	91 aa–96 aa 127 aa–130 aa	55 aa–57 aa 92 aa–94 aa 113 aa–121 aa	43.80 kcal/mol
I-TASSER method and interacting LY6K amino acid residues (aa)	63 aa 70 aa–74 aa 86 aa–89 aa 92 aa	91 aa 93 aa–95 aa	58.85 kcal/mol

## Data Availability

Not applicable.
